# Efficacy of probiotic intervention in unmedicated depression: a systematic review and meta-analysis

**DOI:** 10.3389/fpsyt.2025.1608238

**Published:** 2026-01-07

**Authors:** Liu Haiyan, Wang Dan, Wan Xiaochao, Chen Xiuxiu, Liu Ye, Chen Zhiguo, Liu Ting

**Affiliations:** The 82nd Army Group Hospital of the People's Liberation Army (PLA), Baoding, Hebei, China

**Keywords:** depression, meta-analysis, microbiota-gut-brain axis, probiotics, unmedicated

## Abstract

**Objective:**

To assess the independent efficacy and safety of probiotics in unmedicated adults with depression, with a focus on studies approximating monotherapy conditions.

**Methods:**

This systematic review and meta-analysis followed PRISMA 2020 guidelines and was registered with PROSPERO (CRD420251015474). Four major databases were searched through March 2025 for randomized controlled trials (RCTs) investigating probiotic monotherapy in individuals with depression not receiving psychotropic treatment. All forms of standardized probiotic formulations (e.g., capsules, sachets) were eligible. The primary outcome was the change in validated depression rating scales. Standardized mean differences (SMDs) were synthesized using a random-effects model. Sensitivity and subgroup analyses addressed intervention type, assessment method (self-report vs. clinician-rated scales), and funding source. Safety outcomes were systematically assessed.

**Results:**

Six RCTs with 341 randomly assigned participants (169 probiotic, 172 placebo) were included. Probiotic monotherapy was associated with a small but statistically significant reduction in depressive symptoms (SMD = −0.38, 95% CI: −0.57 to −0.18, p = 0.0002, I² = 51%). Exploratory subgroup analysis indicated potential greater benefit in mild to moderate depression compared to major depressive disorder. Sensitivity analysis excluding industry-funded trials or studies with adjunctive agents resulted in non-significant findings (SMD = −0.21, 95% CI: −0.65 to 0.23, p = 0.35). Minor adverse events were reported, with no significant difference between groups and no serious adverse events.

**Conclusion:**

Probiotic monotherapy may provide modest improvement in depressive symptoms and is generally safe for unmedicated individuals with mild to moderate depression. Given the small effect size, possible industry-related bias, and study heterogeneity, these findings should be interpreted cautiously. Larger, independently-funded RCTs are warranted to confirm efficacy and clarify mechanisms.

**Systematic Review Registration:**

https://www.crd.york.ac.uk/prospero/, iidentifier CRD420251015474.

## 1 Introduction

Depression is a prevalent mental disorder characterized by persistent low mood, diminished interest, and cognitive and physical symptoms, which can severely impair quality of life and function. Over 300 million people worldwide are affected, and depression is projected to become a leading cause of disability by 2030 (1, 2). The disorder imposes significant psychological, social, and economic burdens on individuals and healthcare systems globally.

Current treatment strategies for depression primarily encompass pharmacotherapy, psychotherapy, and physical therapies such as deep brain stimulation and transcranial magnetic stimulation. Antidepressant medications, particularly selective serotonin reuptake inhibitors (SSRIs), constitute the first-line treatment (3). However, treatment response varies considerably, with approximately 30% of patients exhibiting resistance to antidepressants, and many failing to achieve full symptom remission (4–7). Furthermore, medication-related side effects, including nausea, insomnia, and weight fluctuations, often compromise patient adherence (5, 8). While psychotherapy demonstrates efficacy, its widespread implementation is hampered by high costs, limited access to specialized resources, and patient adherence issues. These limitations necessitate the exploration of novel interventions that are safe, effective, and well-tolerated.

The microbiota-gut-brain axis (MGBA) theory has recently emerged, offering a novel perspective on the pathophysiology of depression (9, 10). Research suggests that the underlying mechanisms extend beyond neurotransmitter imbalances to encompass gut microbiota dysbiosis and its impact on the MGBA. Gut microbes influence central nervous system function by modulating neurotransmitter synthesis, immune-inflammatory responses, and intestinal barrier integrity. Individuals with depression exhibit distinct gut microbiota profiles, including reduced abundance of short-chain fatty acid (SCFA)-producing bacteria (e.g., Faecalibacterium and Roseburia) and elevated pro-inflammatory cytokines (e.g., IL-6, TNF-α), correlating with depression severity (11). Animal studies further demonstrate the “transmissibility” of depressive-like behaviors via fecal microbiota transplantation in germ-free mice, while specific probiotic supplementation can reverse hippocampal brain-derived neurotrophic factor (BDNF) downregulation and microglial overactivation (12). These findings provide a compelling rationale for exploring gut microbiota interventions for depression.

Probiotics, recognized for their role in modulating gut microbiota balance, have garnered increasing attention as potential therapeutic agents for depression. By influencing the gut microbiota, mitigating inflammation, enhancing intestinal barrier function, and modulating neurotransmitter synthesis, probiotics may indirectly alleviate depressive symptoms via the MGBA (13–15). Studies suggest that probiotics like Lactobacillus and Bifidobacterium may exert antidepressant effects through several mechanisms: (1) immunomodulation by inhibiting the NF-κB pathway and reducing pro-inflammatory cytokine release; (2) neurotransmitter modulation by promoting the synthesis of gamma-aminobutyric acid (GABA), serotonin, and the dopamine precursor tryptophan; and (3) barrier repair by upregulating tight junction proteins (e.g., Claudin-1, ZO-1) and mitigating lipopolysaccharide (LPS)-induced systemic inflammation (16–19). However, inconsistencies across randomized controlled trials (RCTs), coupled with heterogeneity in probiotic strain, dosage, and intervention duration, hinder systematic evaluation of their efficacy and mechanisms (20, 21).

Furthermore, existing meta-analyses often include studies combining antidepressants with probiotics. Since SSRIs themselves can modulate gut microbiota, this may confound the independent effects of probiotics, leading to ambiguous efficacy attribution (9, 22). Strain specificity, dosage, and treatment duration heterogeneity further limit the generalizability of current findings (23). Addressing these limitations, this study uniquely focuses on RCTs of individuals with depression not receiving psychotropic medications, thereby eliminating the confounding effects of drug-microbiota interactions to provide a comprehensive assessment of probiotics’ independent effects. Moreover, subgroup analyses will explore the influence of intervention duration, depression severity, and measurement scales on treatment outcomes.

This analysis addresses key limitations in existing evidence by focusing specifically on studies of probiotic monotherapy in unmedicated individuals, a design that minimizes confounding by psychotropic drugs. Additionally, we quantitatively explore the potential differential effects of probiotics across depression severity levels, which may inform hypotheses about their utility in early intervention. The goal of this work is to critically appraise the current evidence base for the independent efficacy of probiotics in depression.

In conclusion, depression remains a significant global public health challenge, necessitating the exploration of novel management strategies. This study provides a preliminary assessment of probiotic monotherapy by applying rigorous criteria to minimize confounding. The findings, which indicate a small but significant effect size alongside a favorable safety profile, suggest potential value in specific contexts. However, the limitations—including a small effect, potential for funding bias, and heterogeneous interventions—warrant caution. This work offers a clearer, more focused evidence base that can inform the design of future, definitive trials to conclusively determine the role of probiotics in depression care.

## 2 Materials and methods

### 2.1 Subgroup analysis inclusion and exclusion criteria

Inclusion Criteria:

1. Clinical randomized controlled trials (RCTs);
2. Full-text articles;
3. Adults diagnosed with depression by a psychiatrist;
4. Probiotic intervention (any strain, dosage, formulation, or delivery mode) vs. placebo;
5. No concurrent psychotropic medication use;
6. Outcomes measured using validated depression rating scales (e.g., BDI, PHQ-9, HAMD, MADRS, DASS-D);
7. Sufficient data for meta-analysis.

Exclusion Criteria:

1. Non-clinical, animal, or non-randomized studies;
2. Reviews or protocols;
3. Incomplete data after attempts to contact authors;
4. Inaccessible full text;
5. Non-quantitative or unclear outcome measures.

### 2.2 Search strategy and information sources

This study adhered to the PRISMA 2020 statement. Two researchers independently and systematically searched PubMed, Embase, Cochrane Library, and Web of Science. The search terms included: “Probiotics,” “Depression,” and “trial.” A combination of MeSH terms and free-text words was used for the search strategy, adapting to the specific characteristics of each database. Full search strategies for each database (PubMed, Embase, Cochrane Library, and Web of Science), including database-specific search strings, are provided in **Supplementary Table S1**.

The initial search was conducted from database inception to March 6, 2025.

### 2.3 Study selection and data extraction

Two reviewers independently screened citations, evaluated full texts, and extracted data using a standardized form. Extracted information included study characteristics, population, intervention details, outcome measures, and adverse events. Discrepancies were resolved by consensus with a third reviewer.

### 2.4 Risk of bias assessment

Risk of bias was assessed using the Cochrane Risk of Bias tool, considering random sequence generation, allocation concealment, blinding, incomplete outcome data, selective reporting, and other sources of bias. Studies were graded as low (A), moderate (B), or high(C) risk of bias.

### 2.5 Statistical analysis

Given the heterogeneity of depression rating scales, continuous outcomes were synthesized as standardized mean differences (SMDs) with 95% confidence intervals (CIs). All meta-analyses were performed in Review Manager 5.3. We assessed statistical heterogeneity using the I² statistic and employed a random-effects model if I² > 50%. In cases where individual studies contributed results from multiple depression scales, each scale was treated as an independent comparison in subgroup and sensitivity analyses. This approach was adopted to examine potential scale-specific effects and maximize data use, with the acknowledgment that it introduces statistical non-independence. Sensitivity analyses were conducted by stratifying according to the type of measurement tool (self-rated vs. observer-rated) and funding source. Publication bias was explored through visual inspection of funnel plots; however, this method is unreliable with the inclusion of fewer than ten studies. For the crossover trial (24), data from the first intervention period only were included in the meta-analysis to avoid carry-over effects.

## 3 Results

### 3.1 Study selection

**Figure 1:**
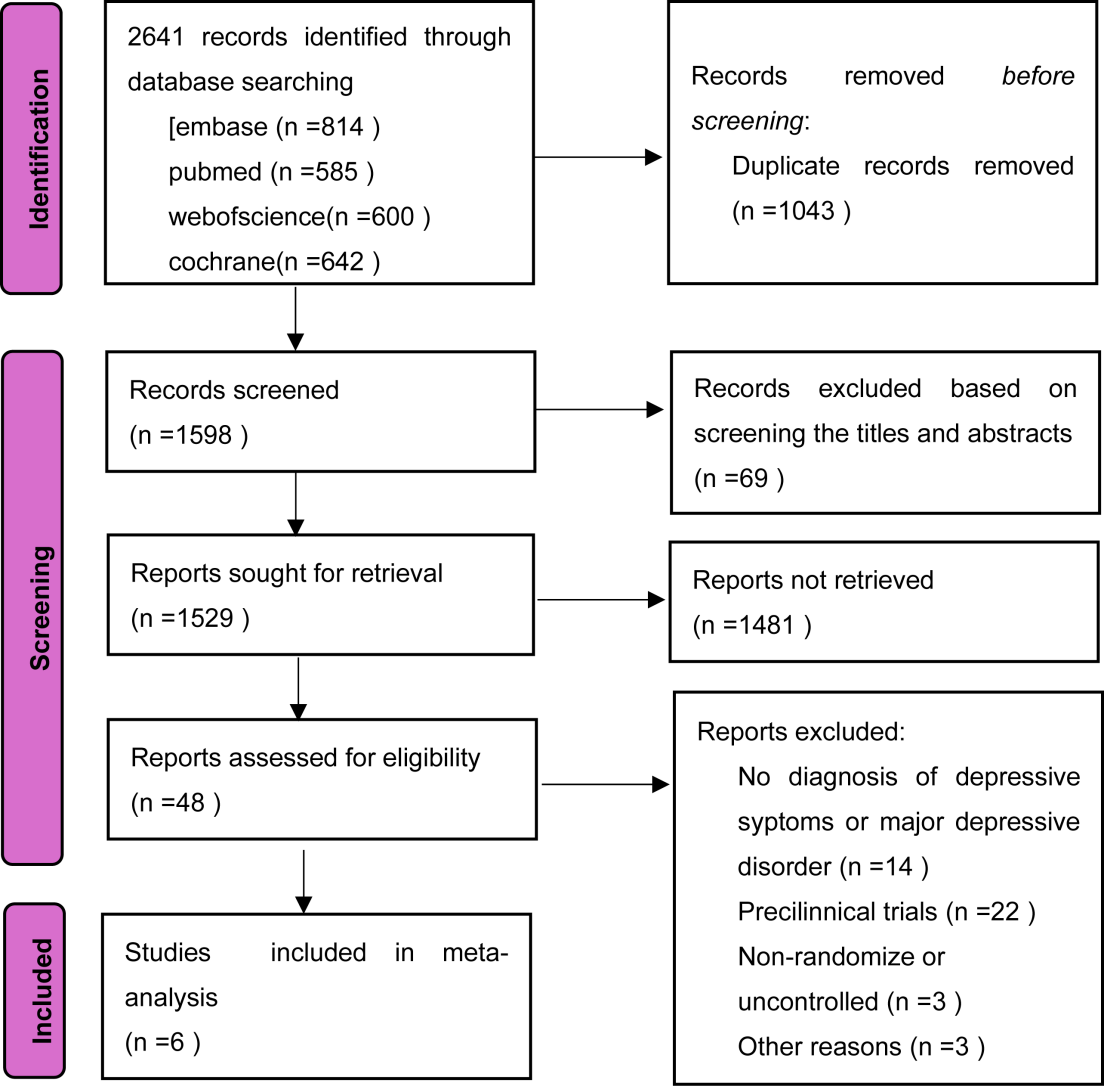
Diagram of the process used to identify references for the review.

The initial search yielded a total of 2641 potentially relevant articles. After removing duplicates using Endnote X9 reference management software and manual verification, 1598 articles remained. Following a review of titles and abstracts, studies with limited relevance to the research question were excluded based on the inclusion and exclusion criteria. The full texts of 48 articles were retrieved for detailed evaluation. After rigorous application of the inclusion and exclusion criteria, 6 studies (24–29) were ultimately included in the meta-analysis (**Figure 1)**.

### 3.2 Characteristics of included studies

Six RCTs enrolling 341 participants (169 probiotic, 172 placebo) were included. Five trials evaluated probiotic monotherapy (Bifidobacterium, Lactobacillus or multi-strain formulations), whereas one trial (24) used a combination of probiotics plus S-adenosyl-methionine. Study-level details are summarised in **Table 1**. Because several trials reported more than one depression scale, the number of measurement-occasions in the forest plots æexceeds the actual number of randomized individuals.

**Table 1:** Characteristics of included studies. ßN = number of participants; SD = standard deviation; Assessment scales include BDI, PHQ-9, HAMD, MADRS, DASS-D.

Author	Country	Year	Design	Age	Depression severity	Probiotics	Duration	Assessment scale	Before probiotic treatment			After probiotic treatment			N1	Before control treatment			After control treatment			N2
									Mean	SD		Mean		SD		Mean		SD	Mean		SD	
Akkasheh.	Iran	2015	Randomized parallel double-blind	20-55	Severe	Lactobacillus acidophilus, Lactobacillus caßßßsei and Bifidobacterium	8W	BDI	Decrease5.7 ± 6.4						17	Decrease1.5 ± 4.8						18
Baião	United Kingdom	2021	Randomized parallel double-blind	18-55	Mild	(Bio-Kult^®^ Advanced, ADM Protexin Ltd)Contains 14 species of bacteria	4W	PHQ-9	12.01	0.76	6.72		0.90		25	12.65	0.75			9.96	0.88	26
Chahwan	Australia	2019	Random parallel triple blind	36.06 ± 12.04	Moderate to Severe	Ecologic^®^BarrierContains 9 species of bacteria	8W	BDI-II	28.91	10.1	19.88		13.44		34	27.97	9.79			19.25	11.96	37
								DASS-depression	22.88	9.96	15.18		14.53		20.43	10.76			12.97	9.34
Majeed	India	2018	Randomized multicenter double-blind	20-65	Severe	B. coagulans MTCC 5856	90d	HAM-D	13.6	4.41	5.9		4.88		20	14.5	3.41			12.5	8.70	20
								MADRS	16.3	5.40	6.0		5.79		17.1	4.63			12.6	8.00
								CES-D	19.1	5.25	8.0		6.17		20.7	4.86			16.7	13.03
Romijn	New Zealand	2017	Randomized parallel-group, double-blind	≧̸ 16	Moderate to Severe	Strain I-1722 in Lactobacillus helveticus R0052, Bifidobacterium longum R0175 (CNCM strain I-3470) bacteria	8W	MADRS	28.3	6.1	19.3		8.9		40	27	6.3			17.6	9.5	39
								QIDS-SR16	15.2	3.9	8.5		5.5		13.2	3.8			8	4.8
								DASS-Depression	24.2	9.1	13.2		11.6		19	10.5			10.3	8.8
Ullah	Italy	2022	randomized, double-blind, crossover	18-65	Mild to Moderate	Lactobacillus rototsetii Rousseti 52, Bifidobacterium longum 175	12W	PHQ-9	8.15	3.28	4.17		2.11		33	7.78	3.44			7.18	3.27	32
							HAMD	11.52	5.48		6.89		4.23			4.17	2.11			10.69	4.49

The intervention in the study by Ullah et al. (24) was a combination of probiotics (Lactobacillus rhamnosus Rosuell S2 and Bifidobacterium longum 175) and S-adenosyl methionine. All other interventions were probiotic monotherapy.

### 3.3 Risk of bias assessment

**Figure 2:**
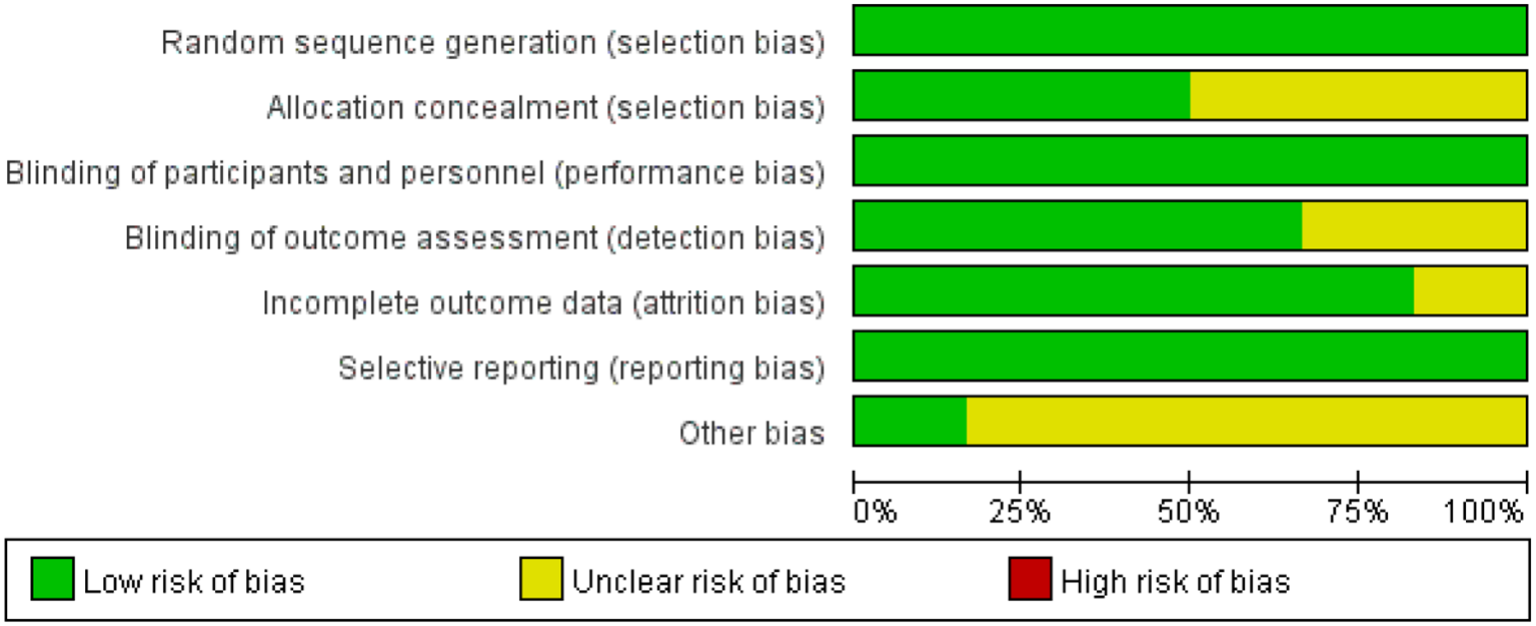
Proportion of projects with risk of bias in included studies.

**Figure 3:**
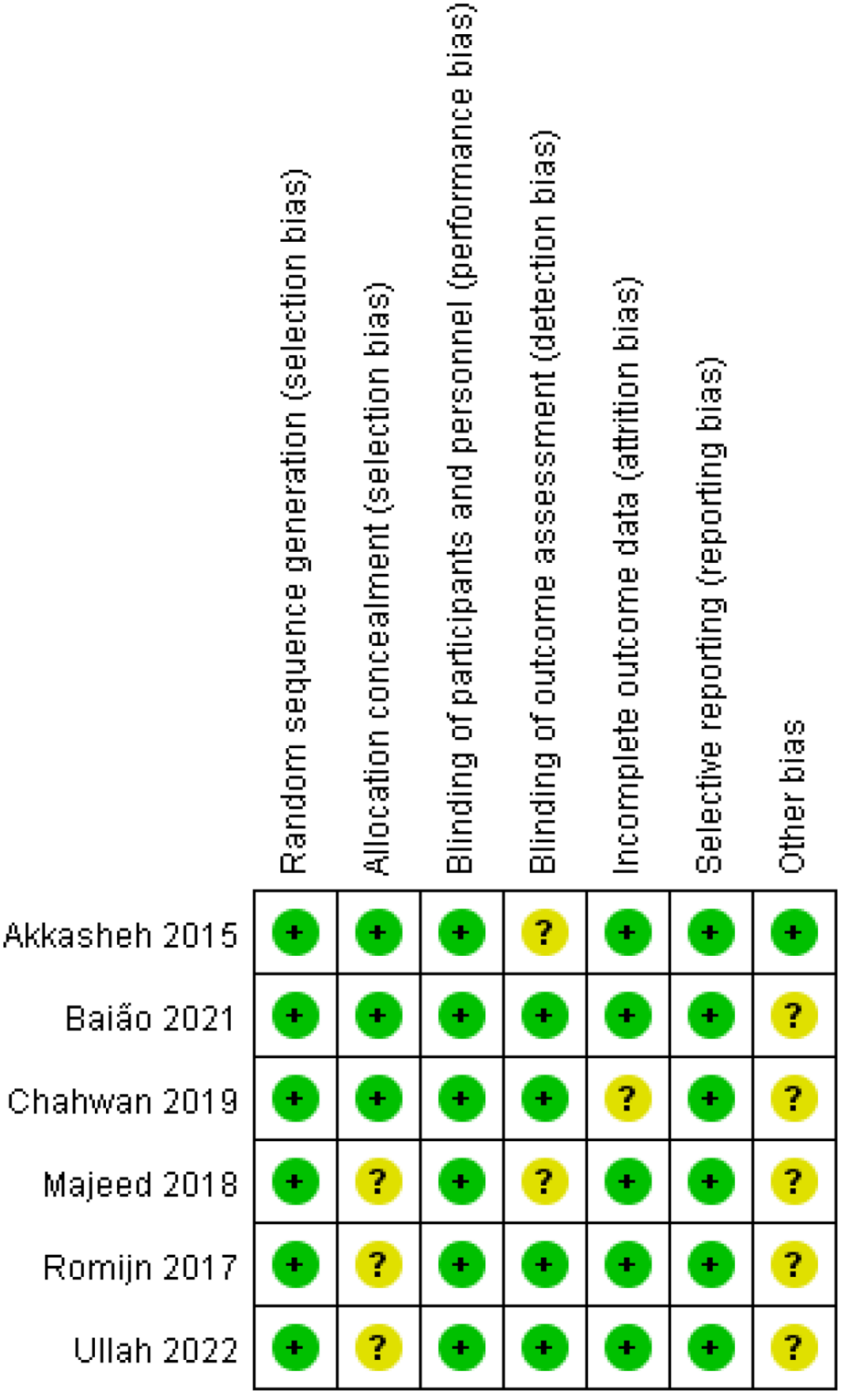
Included literature quality assessment results.

One study was rated as low risk of bias; five were moderate; none were high risk. The main concerns related to incomplete reporting of allocation concealment and selective reporting. The risk of bias assessment is illustrated in **Figures 2**, **3**.

### 3.4 Meta-analysis results

#### 3.4.1 Efficacy of probiotics on depression

**Figure 4:**
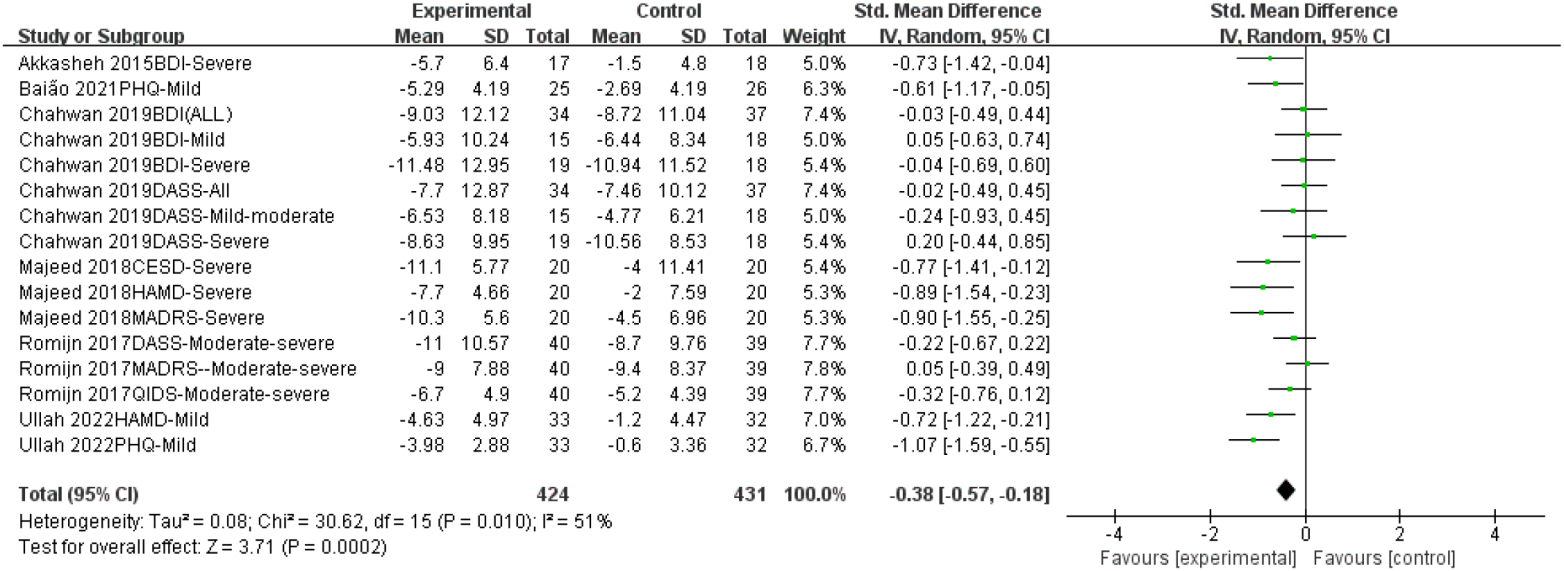
Forest plot of the effect of probiotic monotherapy versus placebo on depressive symptoms across six RCTs. SMD, standardized mean difference; CI, confidence interval; I², measure of heterogeneity.

**Figure 5:**
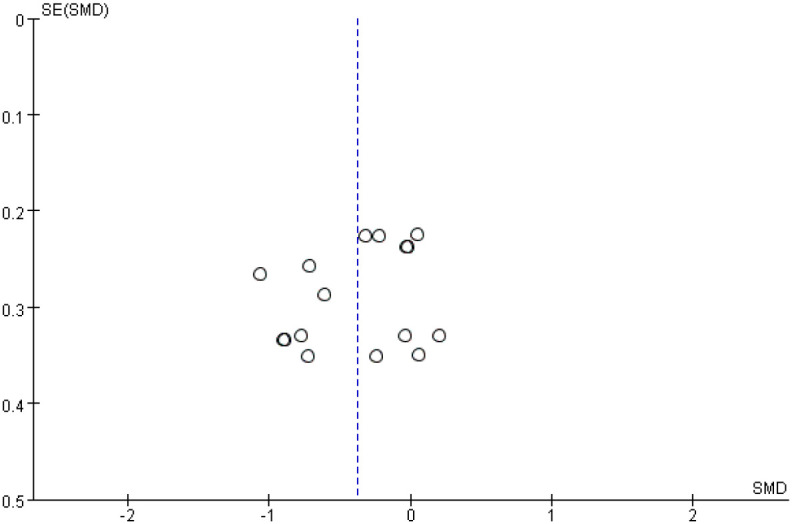
Funnel of publication bias between probiotics and depressive symptoms.

This meta-analysis included six randomized controlled trials (n = 341) to evaluate the efficacy of probiotic monotherapy on depressive symptoms. The random-effects model revealed a significant reduction in depressive symptoms in the probiotic group compared to the control group (Standardized Mean Difference [SMD] = -0.38, 95% Confidence Interval [CI] [-0.57, -0.18], p = 0.0002). A moderate level of heterogeneity was observed among the included studies (I² = 51%), which may be attributed to differences in probiotic strains, study populations, or outcome measures. However, the overall effect size remained significant (**Figure 4**). Funnel plots were generated for visual inspection; however, with fewer than ten studies included, the results are not reliable for assessing publication bias. (**Figure 5**).

### 3.5 Subgroup analysis

#### 3.5.1 Subgroup analysis by depression severity

**Figure 6:**
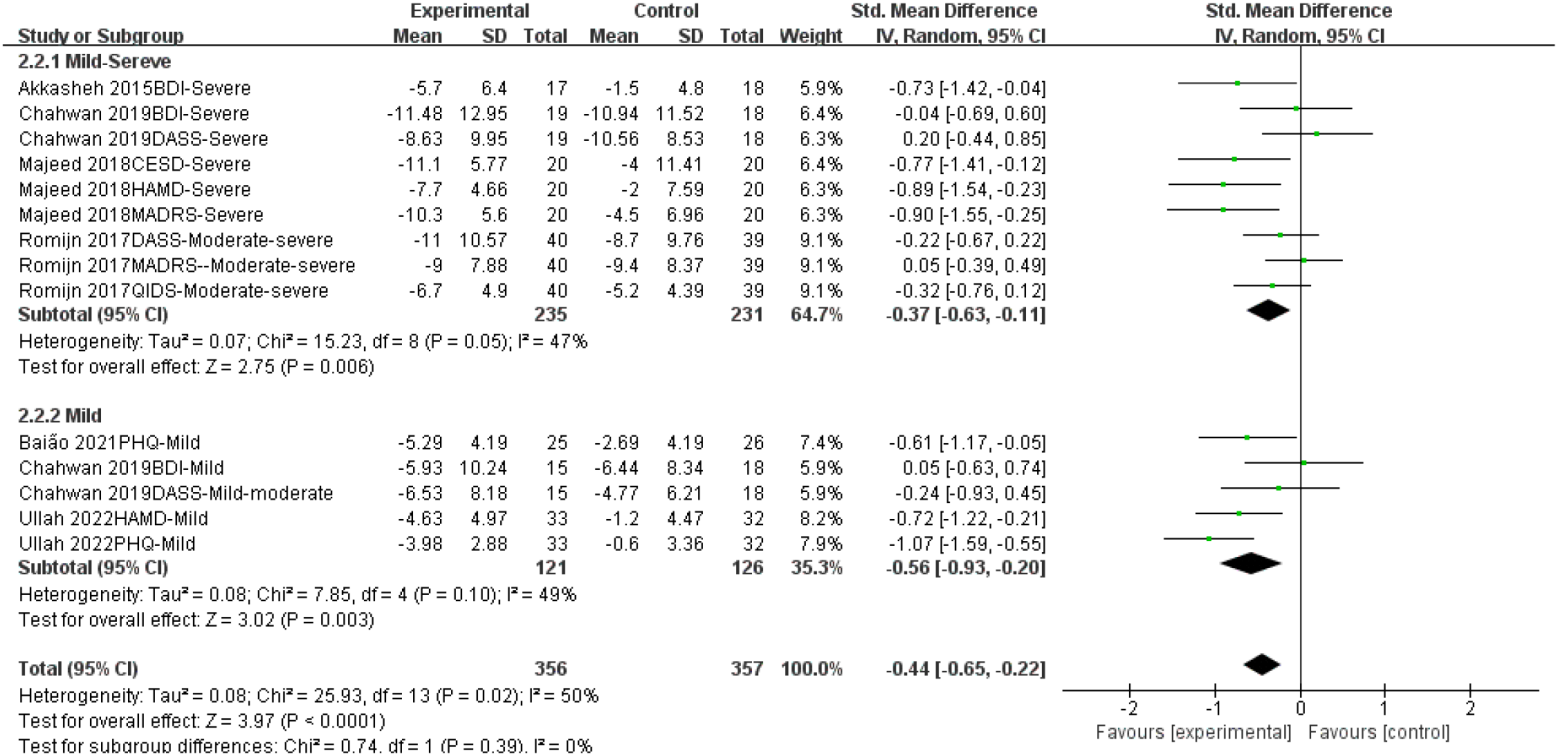
Subgroup analysis of the effect of probiotics on different degrees of depressive symptoms.

Subgroup analyses were performed to explore the differential effects of probiotics by depression severity (mild-to-moderate vs. Severe depression) (**Figure 6**). A total of 9 comparisons (from the six RCTs) were analyzed for Severe depression and 5 for mild-to-moderate depression, as studies using multiple scales contributed a separate comparison for each.

Probiotic supplementation was associated with a significant reduction in depressive symptoms in both Severe depression (SMD = -0.33, 95% CI [-0.52, -0.15], p = 0.0004) and mild-to-moderate depression (SMD = -0.60, 95% CI [-0.86, -0.35], p < 0.00001). The effect size was larger in the mild-to-moderate subgroup.

However, the test for subgroup differences was not statistically significant (Chi² = 2.85, df = 1, p = 0.09; I² = 64.9%). Overall heterogeneity was moderate (I² = 47%). Given the exploratory nature of these analyses, the limited number of studies, and the non-significant subgroup difference, these findings must be interpreted with caution and are considered hypothesis-generating.

#### 3.5.2 Subgroup analysis by psychometric scale type

Overall, probiotic interventions were associated with a significant improvement in depressive symptoms across the scales (SMD = -0.35, 95% CI [-0.49, -0.22], P < 0.0001).

**Figure 7:**
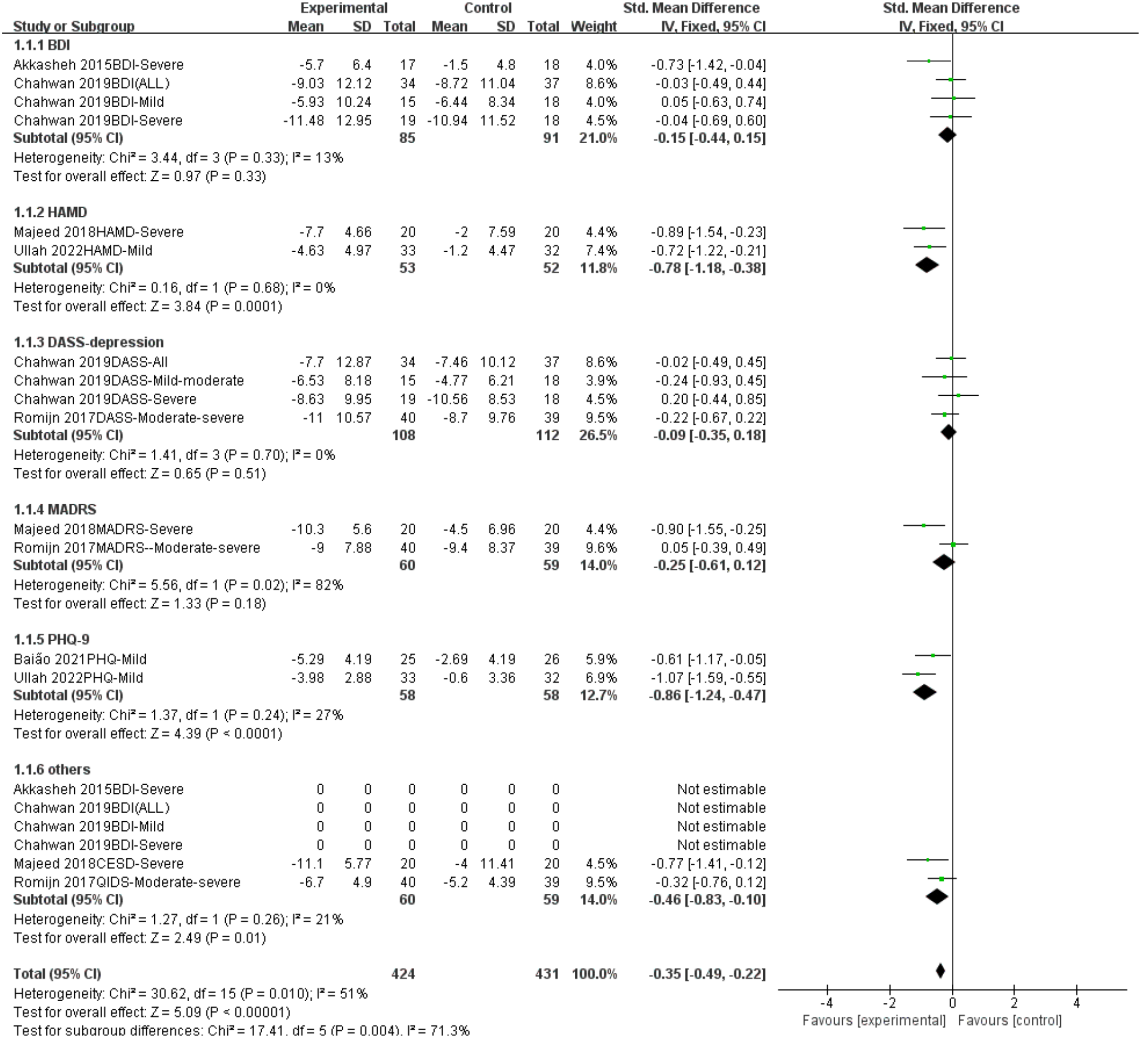
Subgroup analysis of the effect of probiotics on different scales of depression.

Heterogeneity across scales: Variations in effect sizes among different scales may be influenced by probiotic intervention protocols (strains, dosage, combination strategies) and the sensitivity of the scales. Specifically, significant improvements were observed in the Hamilton Depression Rating Scale (HAMD) and Patient Health Questionnaire-9 (PHQ-9), whereas results from the Beck Depression Inventory (BDI), Depression Anxiety Stress Scales - Depression subscale (DASS-depression), and Montgomery-Åsberg Depression Rating Scale (MADRS) did not reach statistical significance. These discrepancies suggest that future studies should optimize strain combinations, increase sample sizes, and further evaluate scale-specific performance differences (**Figure 7**).

### 3.6 Sensitivity analysis

To assess the robustness of the pooled results, we performed a series of sensitivity analyses based on the type of measurement tool and funding source.

#### 3.6.1 Sensitivity analysis by measurement tool

To determine whether the primary conclusion was dependent on a specific category of depression assessment tools, we conducted two sensitivity analyses:

Analysis of Self-Rated Scales Only: When the meta-analysis was restricted to studies using self-rated tools (e.g., BDI, PHQ-9), a significant reduction in depressive symptoms with probiotic monotherapy remained (SMD = -0.31, 95% CI: -0.47 to -0.15, P = 0.0001). Heterogeneity was low-to-moderate (I² = 45%).

Analysis of Observer-Rated Scales Only: When the meta-analysis was restricted to studies using observer-rated tools (e.g., HAMD, MADRS), the intervention also demonstrated a significant and larger effect (SMD = -0.49, 95% CI: -0.76 to -0.22, P = 0.0003), albeit with substantial heterogeneity (I² = 68%).

These analyses indicate that the core finding of efficacy for probiotic monotherapy is robust and not dependent solely on either patient-reported (self-rated) or clinician-assessed (observer-rated) outcomes. The corresponding forest plots are presented in **Supplementary Figures S1** and **S2**.

#### 3.6.2 Sensitivity analysis by funding source

Given that five of the six included trials were industry-funded, we performed a sensitivity analysis by excluding these studies, leaving only the non-industry-funded trial by Akkasheh et al. (25).

After excluding industry-funded trials, the overall effect of probiotic monotherapy was no longer statistically significant (SMD = -0.21, 95% CI: -0.65 to 0.23, p = 0.35). This contrasts with the significant effect observed in the primary meta-analysis and indicates that the positive findings are not robust and are highly dependent on the results from industry-sponsored research. The forest plot for this analysis is presented in **Supplementary Figure S3**.

#### 3.6.3 Sensitivity analysis excluding combination therapy

To assess the robustness of our findings and isolate the effect of probiotic monotherapy, we performed a sensitivity analysis by excluding the study by Ullah et al. (24). As noted in **Table 1**, this study administered a combination of probiotics and S-adenosyl methionine, unlike the pure monotherapy interventions in the other included trials.

After removal of this study, the pooled standardized mean difference (SMD) was attenuated but remained statistically significant (–0.27, 95% CI –0.41 to –0.12, p = 0.0004). It is noteworthy that the heterogeneity among studies substantially decreased from 51% in the primary analysis to 35%, indicating greater consistency in the results when the combination therapy was excluded. Although the overall effect size was slightly attenuated compared to the primary analysis (SMD = -0.35), this sensitivity analysis confirms that the significant beneficial effect of probiotics is robust and not solely dependent on the inclusion of studies containing adjunctive antidepressant components, as evidenced by the persistent beneficial effect even after excluding the study that used a probiotic-S-adenosyl methionine combination [24] (see **Table 1** Note). This exclusion also yielded a more consistent and reliable evidence base, as reflected in substantially reduced heterogeneity (I² = 35%). The forest plot for this analysis is presented in **Supplementary Figure S4**.

### 3.7 Adverse events and safety

Adverse events were systematically monitored in all six included trials. No serious adverse events or withdrawals due to adverse effects were reported in any study. A detailed summary of all minor adverse events, including incidence rates and between-group statistical comparisons for each study, is provided in **Supplementary Table S3**.

Pooled analysis of the five studies that provided complete numerical data (excluding 24) revealed no significant difference in the overall incidence of any adverse event between the probiotic and placebo groups (49.1% vs. 52.0%, χ² = 0.28, p = 0.60). Although a few individual studies reported statistically significant differences for specific adverse events (e.g., somnolence, dry mouth), these findings were not consistent across the included trials.

In conclusion, probiotic monotherapy demonstrated a favorable safety and tolerability profile, with the overall incidence and nature of adverse events being comparable to placebo.

## 4 Discussion

This systematic review and meta-analysis assessed the independent effects of probiotic monotherapy by excluding studies involving participants on psychotropic medications. While this represents a methodological refinement, our findings should be contextualized within the existing literature. The meta-analysis demonstrated a statistically significant reduction in depressive symptoms associated with probiotic supplementation (SMD = -0.38, 95% CI [-0.57, -0.18], p = 0.0002). However, this represents a small effect size, and the clinical relevance of this magnitude of improvement remains uncertain.

The consistency of the primary finding was supported by sensitivity analyses restricted to either self-rated or observer-rated scales. While the effect size varied, the convergent findings across assessment modalities are congruent with a potential biological effect mediated via the gut-brain axis. It is critical to note that mechanistic explanations—including effects on NF-κB signaling, BDNF expression, and microglial activity—are hypothetical and were not directly assessed in the included clinical trials; their biological plausibility is derived from preclinical studies.

Several important limitations must be acknowledged. First, considerable heterogeneity existed in probiotic strains, formulations, and dosages. Second, the included studies had relatively small sample sizes and short-term durations. Most importantly, our sensitivity analysis revealed that the overall effect was no longer statistically significant after the exclusion of industry-funded studies (SMD = -0.21, p = 0.35). This underscores a substantial potential for funding-related bias and necessitates independent verification. Furthermore, the exploratory subgroup analyses were limited by the number of studies and should be considered hypothesis-generating.

Furthermore, our exploratory subgroup analysis revealed a gradient of effect sizes, with a larger benefit observed in mild-to-moderate depression compared to Severe depression. Although the subgroup difference was not statistically significant, this pattern aligns with the hypothesis that modulating the microbiota-gut-brain axis may be more efficacious in the earlier phases of depression. It is plausible that before the neurobiology of severe depression becomes entrenched, interventions like probiotics have a greater capacity to exert a beneficial effect. This hypothesis, generated from our data, clearly highlights a limitation in the current literature—the lack of large-scale trials specifically powered to test the efficacy of probiotics in early intervention—and pinpoints a crucial direction for future research.

Sensitivity analyses evaluated the robustness of the primary finding. The beneficial effect of probiotics persisted even after excluding the study that used a probiotic-S-adenosyl methionine combination [24], yielding a more consistent and reliable evidence base for the independent effect of probiotics, as reflected in substantially reduced heterogeneity. The observed attenuation of the effect size, however, suggests that the inclusion of combination therapies in the broader literature may lead to an overestimation of efficacy for probiotics alone.

Regarding safety, minor adverse events were reported with similar frequency between intervention and control groups, and no serious adverse events were noted, supporting a favorable short-term safety profile.

In conclusion, while probiotic monotherapy may offer modest improvements in depressive symptoms for unmedicated individuals, the effect size is small and subject to potential bias. These findings should be interpreted cautiously, and do not establish probiotics as a first-line intervention for depression. Future large, independently-funded randomized controlled trials are required to confirm these results and clarify underlying mechanisms.

All authors declare no conflict of interest.

## Data Availability

The raw data supporting the conclusions of this article will be made available by the authors, without undue reservation.
