# Supplementary material for: Efficacy of probiotic intervention in unmedicated depression: a systematic review and meta-analysis
Source: Front Psychiatry. 2026 Jan 7;16:1608238. doi: 10.3389/fpsyt.2025.1608238 (PMC12819584; doi:10.3389/fpsyt.2025.1608238)

**Supplementary Table S1**: PICO-S Framework.

| Component | Description |
| --- | --- |
| Population (P) | Clinically diagnosed individuals with depression |
| Intervention (I) | Probiotics alone |
| Comparison (C) | Placebo without probiotics |
| Outcome (O) | Efficacy measurement: Changes in depression scale scores from baseline to follow-up, including but not limited to: Beck Depression Inventory (BDI), Hamilton Depression Rating Scale (HAM-D), Montgomery–Åsberg Depression Rating Scale (MADRS) |
| Study Type (S) | Randomized Controlled Trial (RCT) |

**Supplementary Table S2**: Full electronic search strategy across all databases:

| Database | Platform/Interface | Search Date | Search Query | Results |
| --- | --- | --- | --- | --- |
| PubMed | https://pubmed.ncbi.nlm.nih.gov/ | 6-Mar-25 | #1 Depression[Title/Abstract] OR Depressive Symptoms[Title/Abstract] OR Depressive Symptom[Title/Abstract] OR "Symptom, Depressive"[Title/Abstract] OR "Emotional Depression"[Title/Abstract] OR "Depression, Emotional"[Title/Abstract] OR "Depressive Disorders"[Title/Abstract] OR "Disorder, Depressive"[Title/Abstract] OR "Disorders, Depressive"[Title/Abstract] OR "Neurosis, Depressive"[Title/Abstract] OR "Depressive Neuroses"[Title/Abstract] OR "Depressive Neurosis"[Title/Abstract] OR "Neuroses, Depressive"[Title/Abstract] OR "Depression, Endogenous"[Title/Abstract] OR "Depressions, Endogenous"[Title/Abstract] OR "Endogenous Depression"[Title/Abstract] OR "Endogenous Depressions"[Title/Abstract] OR Melancholia[Title/Abstract] OR Melancholias[Title/Abstract] OR "Unipolar Depression"[Title/Abstract] OR "Depression, Unipolar"[Title/Abstract] OR "Depressions, Unipolar"[Title/Abstract] OR "Unipolar Depressions"[Title/Abstract] OR "Depressive Syndrome"[Title/Abstract] OR "Depressive Syndromes"[Title/Abstract] OR "Syndrome, Depressive"[Title/Abstract] OR "Syndromes, Depressive"[Title/Abstract] OR "Depression, Neurotic"[Title/Abstract] OR "Depressions, Neurotic"[Title/Abstract] OR "Neurotic Depression"[Title/Abstract] OR "Neurotic Depressions"[Title/Abstract] #2 Probiotic[Title/Abstract] OR probiotics[Title/Abstract] OR bifidobacterium[Title/Abstract] OR lactobacillus[Title/Abstract] OR bacillus[Title/Abstract] OR yeast[Title/Abstract] OR bacteriotherapy[Title/Abstract] #3 randomized controlled trial[Title/Abstract] OR "Clinical Trials"[Title/Abstract] OR Randomized[Title/Abstract] OR Trials[Title/Abstract] OR "Randomized Clinical"[Title/Abstract] OR "Controlled Clinical Trials"[Title/Abstract] OR "random allocation"[Title/Abstract] OR "Allocation,Random"[Title/Abstract] OR Randomization[Title/Abstract] OR "double-blind"[Title/Abstract] OR randomly[Title/Abstract] OR RCT[Title/Abstract] OR random*[Title/Abstract] #4 #1 AND #2 AND #3 | 585 |
| Embase | Ovid | 4-Mar-25 | #1 'depression'/exp OR depression OR 'depressive symptoms':ab,ti OR 'depressive symptom':ab,ti OR 'symptom, depressive':ab,ti OR 'emotional depression':ab,ti OR 'depression, emotional':ab,ti OR 'depressive disorders':ab,ti OR 'disorder, depressive':ab,ti OR 'disorders, depressive':ab,ti OR 'neurosis, depressive':ab,ti OR 'depressive neuroses':ab,ti OR 'depressive neurosis':ab,ti OR 'neuroses, depressive':ab,ti OR 'depression, endogenous':ab,ti OR 'depressions, endogenous':ab,ti OR 'endogenous depression':ab,ti OR 'endogenous depressions':ab,ti OR melancholia:ab,ti OR melancholias:ab,ti OR 'unipolar depression':ab,ti OR 'depression, unipolar':ab,ti OR 'depressions, unipolar':ab,ti OR 'unipolar depressions':ab,ti OR 'depressive syndrome':ab,ti OR 'depressive syndromes':ab,ti OR 'syndrome, depressive':ab,ti OR 'syndromes, depressive':ab,ti OR 'depression, neurotic':ab,ti OR 'depressions, neurotic':ab,ti OR 'neurotic depression':ab,ti OR 'neurotic depressions':ab,ti #2 'probiotic'/exp OR probiotic OR probiotics:ab,ti OR bifidobacterium:ab,ti OR lactobacillus:ab,ti OR bacillus:ab,ti OR yeast:ab,ti OR bacteriotherapy:ab,ti #3 'randomized controlled trial'/exp OR 'randomized controlled trial' OR (randomized AND controlled AND ('trial'/exp OR trial)) OR 'clinical trials':ab,ti OR 'randomized controlled trial':ab,ti OR trials:ab,ti OR 'randomized clinical':ab,ti OR 'controlled clinical trials':ab,ti OR randomized:ab,ti OR 'random allocation':ab,ti OR 'allocation,random':ab,ti OR randomization:ab,ti OR 'double blind':ab,ti OR randomly:ab,ti OR rct:ab,ti OR random*:ab,ti #4 #1 AND #2 AND #3 | 814 |
| Web of Science | Web of Science Core Collection | 4-Mar-25 | #119 TS=(Depression) OR AB=(Depressive Symptoms OR Depressive Symptom OR "Symptom, Depressive" OR "Emotional Depression" OR "Depression, Emotional" OR "Depressive Disorders" OR "Disorder, Depressive" OR "Disorders, Depressive" OR "Neurosis, Depressive" OR "Depressive Neuroses" OR "Depressive Neurosis" OR "Neuroses, Depressive" OR "Depression, Endogenous" OR "Depressions, Endogenous" OR "Endogenous Depression" OR "Endogenous Depressions" OR Melancholia OR Melancholias OR "Unipolar Depression" OR "Depression, Unipolar" OR "Depressions, Unipolar" OR "Unipolar Depressions" OR "Depressive Syndrome" OR "Depressive Syndromes" OR "Syndrome, Depressive" OR "Syndromes, Depressive" OR "Depression, Neurotic" OR "Depressions, Neurotic" OR "Neurotic Depression" OR "Neurotic Depressions") #121 TS=(Probiotic) OR AB=(probiotics OR bifidobacterium OR lactobacillus OR bacillus OR yeast OR bacteriotherapy) #122 TS=("randomized controlled trial") OR AB=("Clinical Trials" OR Randomized OR Trials OR "Randomized Clinical" OR "Controlled Clinical Trials" OR "random allocation" OR "Allocation,Random" OR Randomization OR "double-blind" OR randomly OR RCT OR random*) #123 #119 AND #121 AND #122 Refined by: [excluding] Preprint Citation Index | 600 |
| Cochrane Library | Central Register of Controlled Trials | 4-Mar-25 | #1 MeSH descriptor: [Depression] explode all trees #2 (Depression OR "Emotional Depression" OR "Depression, Emotional" OR "Depressive Symptom" OR "Symptom, Depressive" OR "Depressive Symptoms") in Title, Abstract, or Keywords #3 #1 OR #2 #4 MeSH descriptor: [Probiotics] explode all trees #5 (Probiotic or probiotics or bifidobacterium or lactobacillus or bacillus or yeast or bacteriotherapy) in Title, Abstract, or Keywords #6 #4 OR #5 #7 #3 AND #6 | 648 |

Supplementary Table S3: Detailed Summary of Adverse Events from Included Randomized Controlled Trials

| **Study (Author, Year)** | **Adverse Event** | **Probiotics (n/N, %)** | **Placebo (n/N, %)** | **Statistical Test** | **p-value** |
| --- | --- | --- | --- | --- | --- |
| **Akkasheh 2016** | Any Adverse Event | 6/20 (30%) | 5/20 (25%) | χ² | 0.74 |
|  | Flatulence | 3/20 (15%) | 2/20 (10%) | Fisher's Exact | 1 |
|  | Bloating | 2/20 (10%) | 1/20 (5%) | Fisher's Exact | 1 |
|  | Headache | 1/20 (5%) | 2/20 (10%) | Fisher's Exact | 1 |
| **Baiao 2022** | Self-reported Side Effects | 2/35 (5.7%) | 2/36 (5.6%) | Fisher's Exact | 1 |
| **Chahwan 2019** | Any Adverse Event | 23/34 (68%) | 25/37 (68%) | χ² | 1 |
|  | Nausea | 11/34 (32%) | 9/37 (24%) | χ² | 0.42 |
|  | Somnolence | 7/34 (21%) | 2/37 (5%) | χ² | **0.042*** |
|  | Dry Mouth | 2/34 (6%) | 5/37 (14%) | Fisher's Exact | 0.27 |
|  | Dehydration | 3/34 (9%) | 9/37 (24%) | χ² | 0.088 |
| **Majeed 2018** | Fever/Fatigue | 0/20 (0%) | 1/20 (5%) | Fisher's Exact | 1 |
| **Romijn 2017** | Any Adverse Event | 22/40 (55%) | 28/39 (72%) | χ² | 0.12 |
|  | Dry Mouth | 2/40 (5%) | 8/39 (21%) | Fisher's Exact | **0.048*** |
|  | Sleep Disturbance | 0/40 (0%) | 5/39 (13%) | Fisher's Exact | **0.030*** |
|  | Constipation | 7/40 (18%) | 11/39 (28%) | χ² | 0.29 |
|  | Appetite Change | 7/40 (18%) | 10/39 (26%) | χ² | 0.4 |
|  | Nausea | 6/40 (15%) | 7/39 (18%) | χ² | 0.71 |
| **Ullah 2022** | Specific Events | Not Reported | Not Reported | Not Reported | N/A^a^ |
|  | Overall Comparison | Not Reported | Not Reported | Not Significant^b^ | N/A^a^ |

Note: *p* < 0.05. Events marked with an asterisk (*) were statistically significant within the individual study but were not consistent across the body of evidence.

^a^ N/A = Not Applicable. The study by Ullah et al. (2022) did not provide numerical data on the incidence of specific adverse events.

^b^The authors stated in the text that no significant differences in adverse events were observed between groups, but no statistical values (e.g., p-value) were provided.

Supplementary Figure S1:


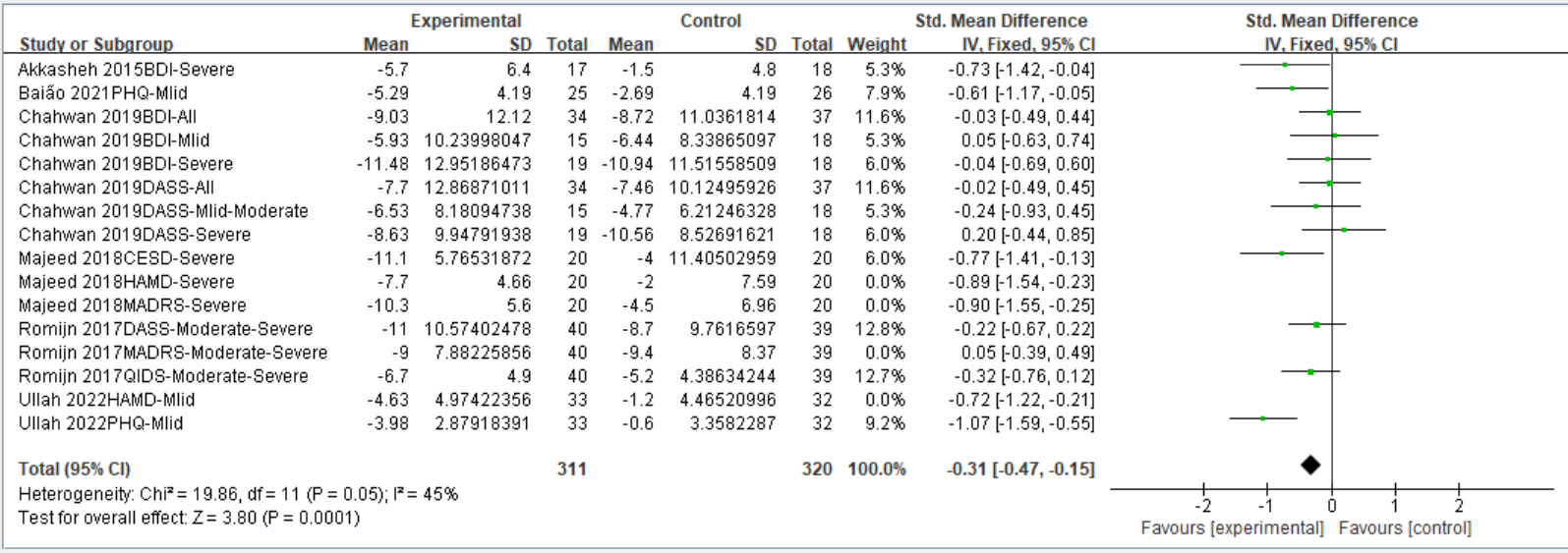


Supplementary Figure S2:


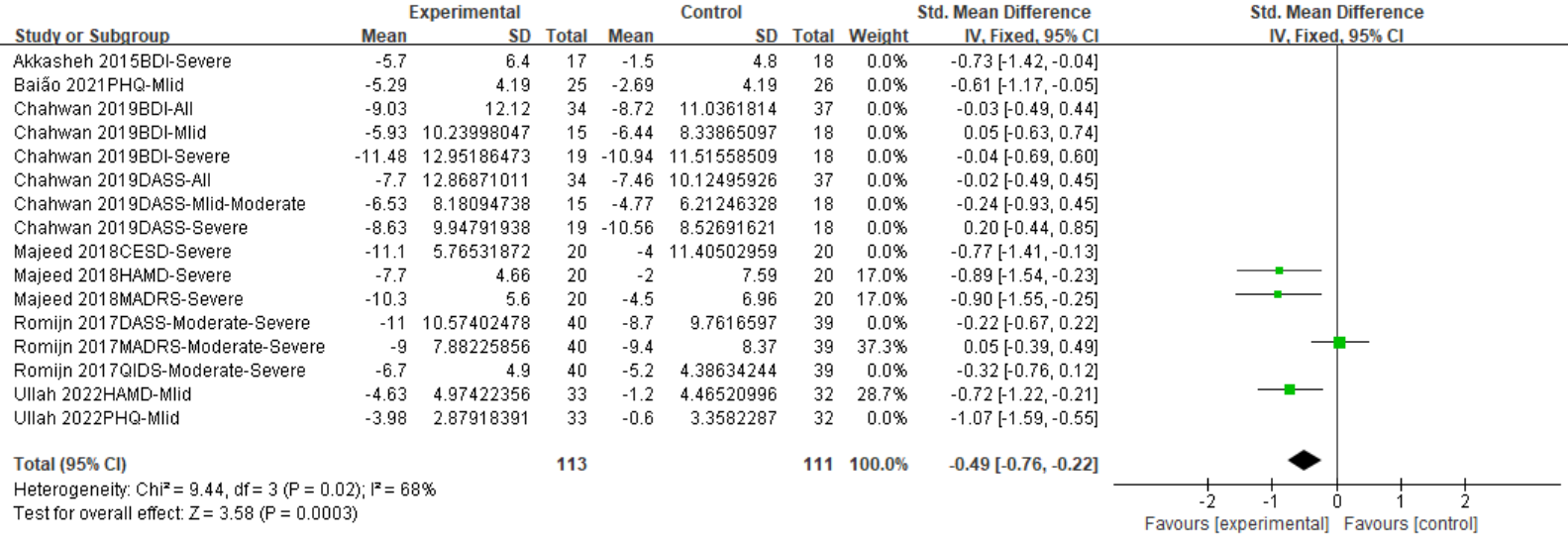


Supplementary Figure S3:


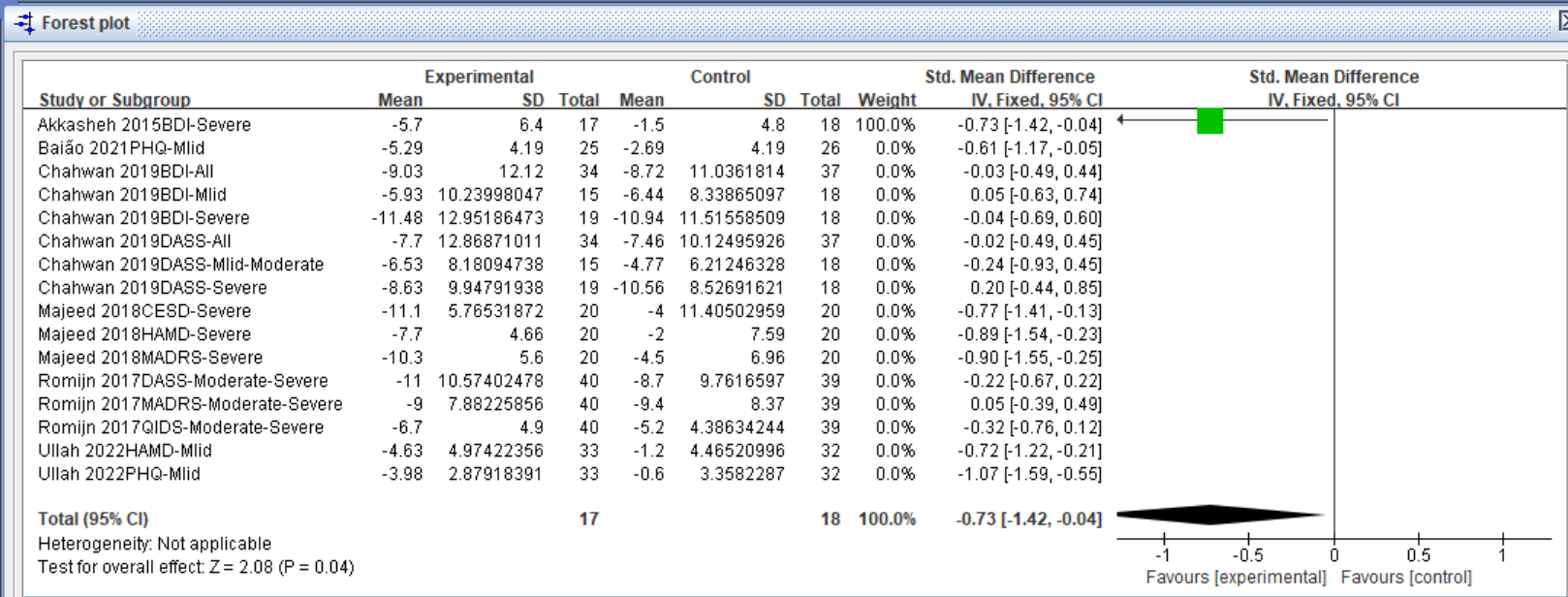


Supplementary Figure S4:


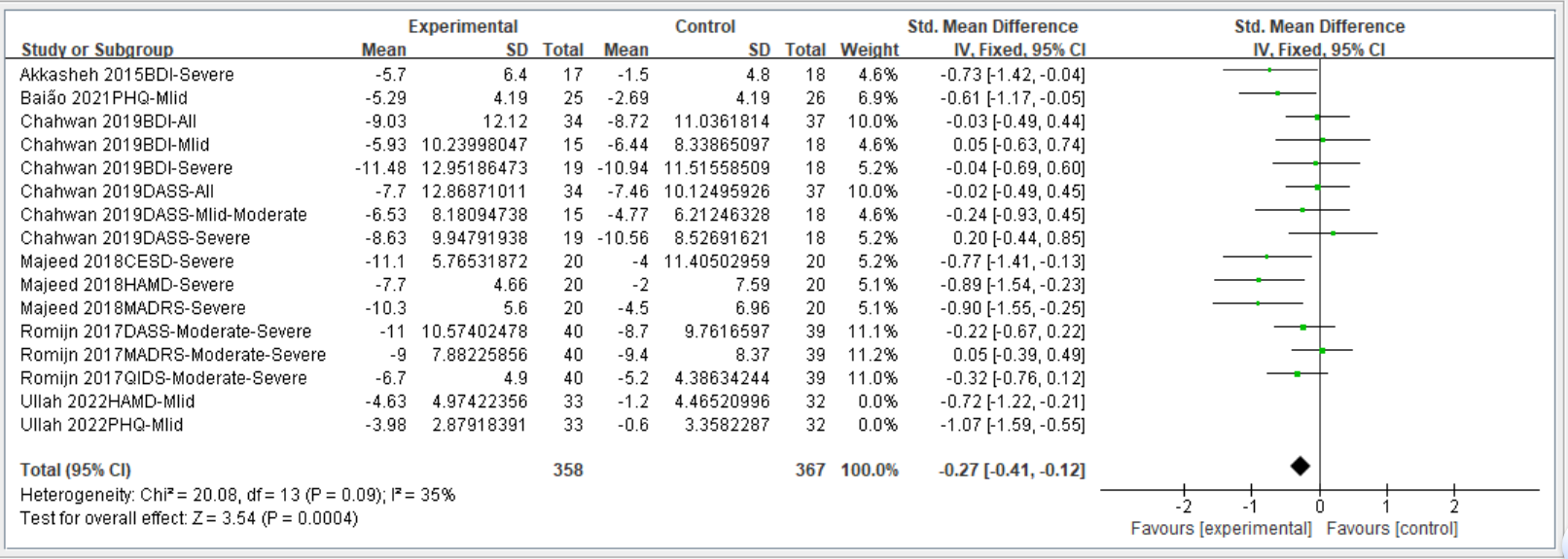

Supplement: Supplementary file 1 [file Table1.doc]
